# Efficient and Site-Specific Incorporation of 3-Nitro-Tyrosine Into Recombinant Proteins in *Escherichia coli*

**DOI:** 10.21769/BioProtoc.5674

**Published:** 2026-04-20

**Authors:** Sarah B. McGee, Stanislau Stanisheuski, Ryan A. Mehl, Richard B. Cooley

**Affiliations:** 1 Department of Biochemistry and Biophysics, Oregon State University, 2011 Agricultural and Life Sciences, Corvallis, OR, USA; 2 GCE4All Research Center, Oregon State University, 2011 Agricultural and Life Sciences, Corvallis, OR, USA

**Keywords:** 3-nitro-tyrosine, Genetic code expansion, Amber suppression, Aminoacyl tRNA synthetase, *Methanocaldococcus jannaschii*, *Methanomethylophilus alvus*

## Abstract

3-nitro-tyrosine (nitroTyr) is one of numerous oxidative protein modifications implicated in diseases such as cardiovascular disease, cancer, and amyotrophic lateral sclerosis (ALS). Because of this, the ability to site-specifically encode nitroTyr into recombinant proteins is a powerful approach for studying these disease pathways. However, producing proteins with defined nitration sites is technically challenging due to the limitations of traditional chemical nitration via peroxynitrite, which lacks residue and site-specificity. Genetic code expansion (GCE) offers a solution by enabling precise incorporation of nitroTyr at designated TAG codons using engineered aminoacyl-tRNA synthetase/tRNA pairs from *Methanocaldococcus jannaschii* and *Methanomethylophilus alvus*. This protocol provides a reliable, optimized workflow for incorporating nitroTyr into proteins in *E. coli* using GCE. It guides users through key considerations in selecting cell lines, media conditions, and GCE systems to minimize off-target effects such as release factor 1 competition, near-cognate suppression, and chemical reduction of nitroTyr. The method is demonstrated using wild-type and TAG-containing superfolder GFP but is broadly applicable to other proteins of interest.

Key features

• This protocol offers a practical guide for the recombinant expression of proteins containing site-specific 3-nitro-tyrosine in *E. coli.*

• These methods should be used to characterize the functional and structural consequences of site-specific tyrosine nitration on proteins without having to modify any other residues.

• This protocol avoids the use of peroxynitrite as a method to nitrate proteins, which modifies all solvent accessible tyrosine residues to different extents.

• Users are guided through the advantages and disadvantages of using different expression strains and genetic code expansion systems depending on specific needs.

## Graphical overview


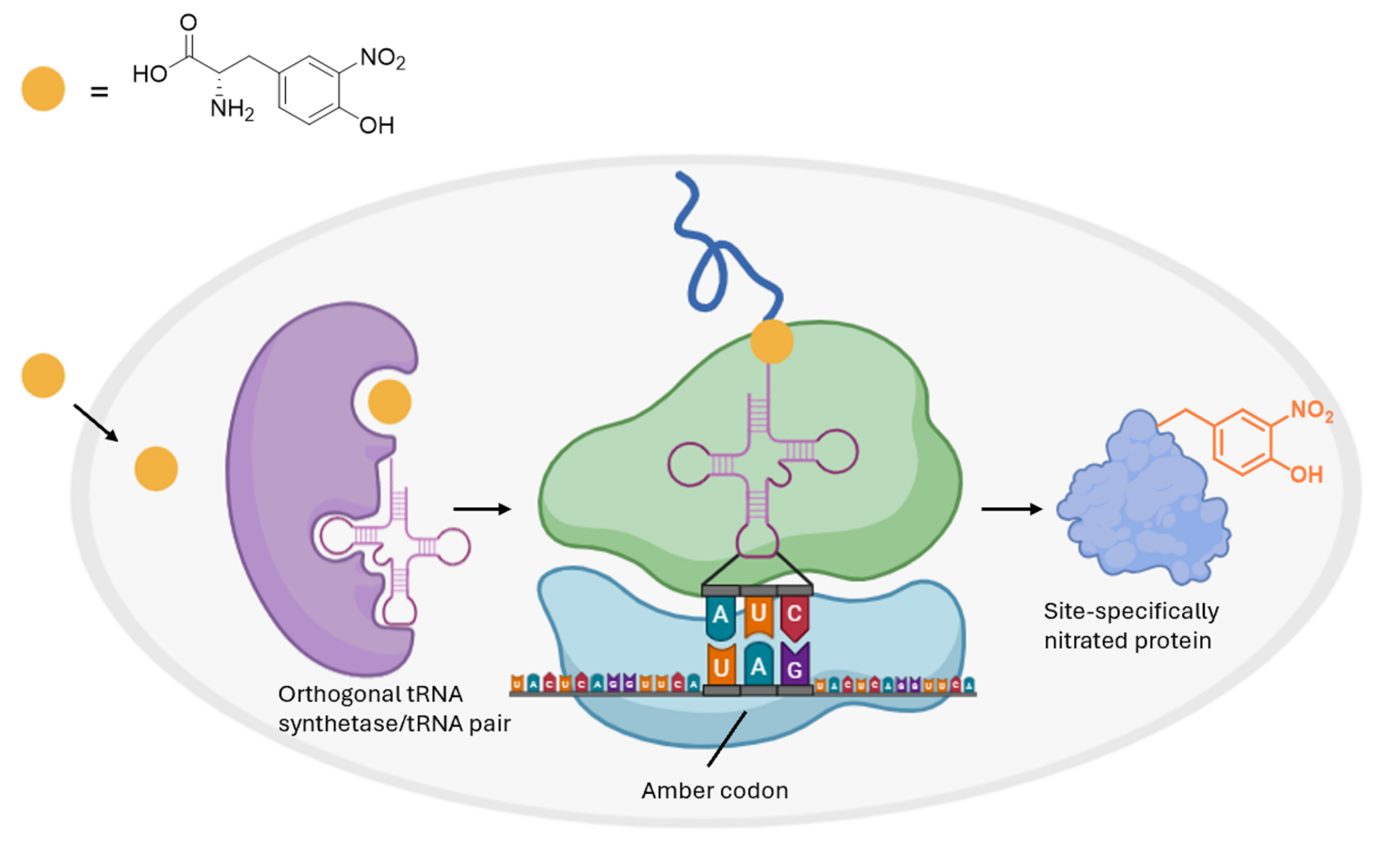


## Background

The accumulation of oxidatively modified proteins is a hallmark of many human diseases, including cardiovascular disease, neurodegeneration, and cancer [1–3]. A key contributor to this phenomenon is oxidative stress, where cells experience an imbalance between oxidants and antioxidants, which leads to cellular damage via reactive oxygen and nitrogen species. Among these, peroxynitrite is a potent oxidant that reacts with tyrosine residues to form 3-nitro-tyrosine (nitroTyr) [4], a stable and commonly detected post-translational modification. Due to its strong association with pathological conditions, nitroTyr has emerged as a widely used marker of oxidative stress in both clinical and research settings [5].

Nitration of tyrosine residues is increasingly recognized as a mechanism that can alter protein function and contribute to disease phenotypes [1–3,6–7]. Several studies have shown that nitration can enhance or inhibit protein activity, affect subcellular localization, or modulate protein–protein interactions [8,9]. In cases where nitroTyr has been introduced into proteins site-specifically, they have been instrumental in confirming that specific nitration events are sufficient to cause gain-of-function and loss-of-function phenotypes in cells, providing causal evidence for the role of nitration in disease mechanisms [10,11].

**Figure 1.**
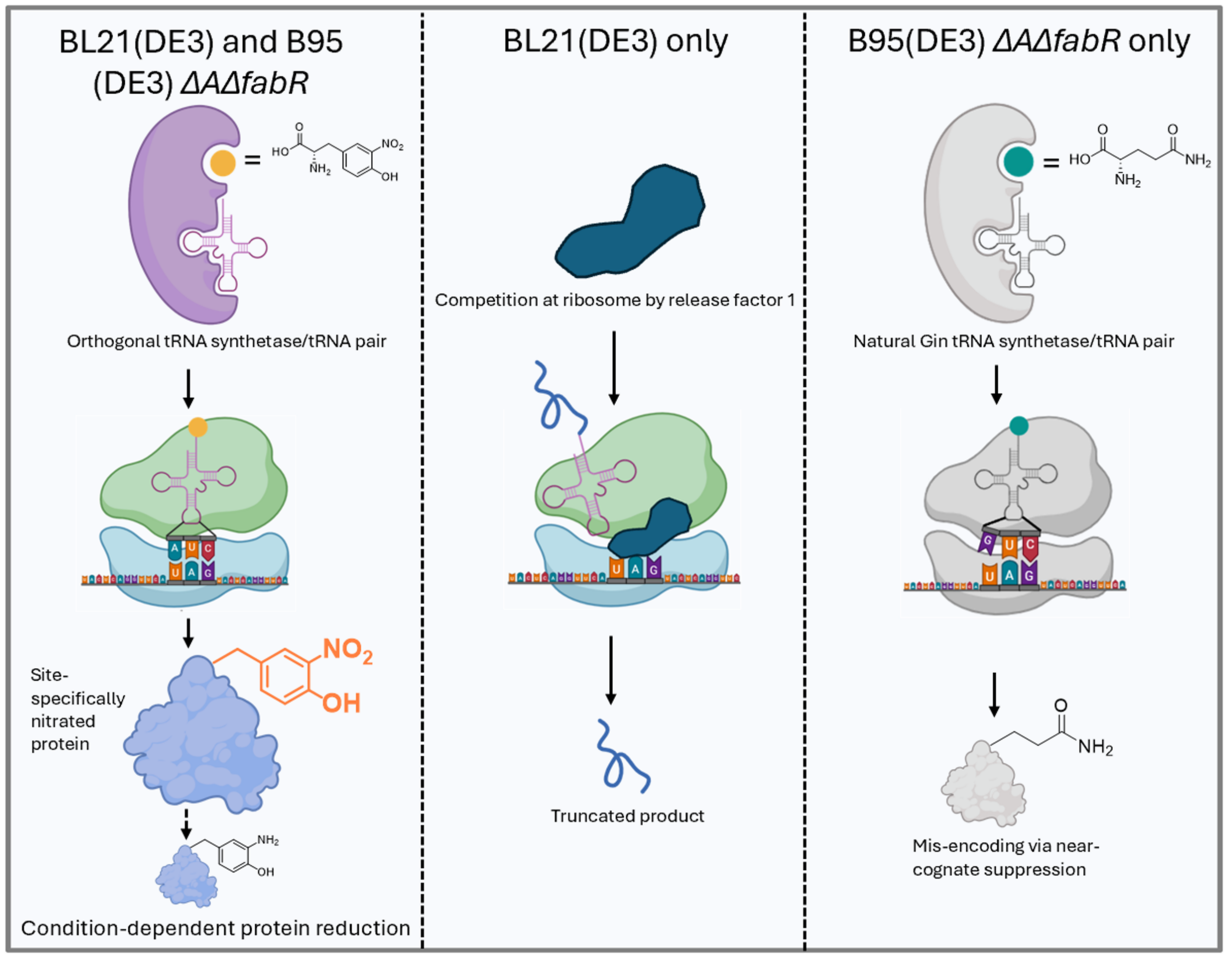
Genetic encoding of nitroTyr in BL21(DE3) and B95(DE3) Δ*A*Δ*fabR* cells. (Left) After entering the cell, nitroTyr is aminoacylated onto its cognate amber-suppressing tRNA by the nitroTyr-specific tRNA-synthetase. At the ribosome, this charged tRNA base pairs with the UAG codon in the mRNA of the protein of interest, resulting in site-specific incorporation of nitroTyr into the growing polypeptide. Under reducing conditions or limited aeration, nitroTyr incorporated into the protein can be chemically reduced to amino-tyrosine. (Middle) In BL21(DE3) cells, which contain release factor 1 (RF1), translation can terminate prematurely at the UAG codon, producing truncated protein in addition to full-length, nitrated protein. (Right) RF1-deficient B95(DE3) Δ*A*Δ*fabR* cells prevent truncation, but inefficient aminoacylation of the nitroTyr-tRNA can lead to near-cognate suppression, where glutamine is mis-incorporated at the UAG codon by endogenous Gln-tRNA.

Genetic code expansion (GCE) allows for this translational incorporation of nitroTyr at genetically defined sites within a protein of interest (Figure 1). This is achieved by repurposing the TAG amber stop codon to encode nitroTyr, using engineered orthogonal aminoacyl-tRNA synthetase/tRNA pairs that recognize nitroTyr. This contrasts with traditional chemical nitration methods, such as treating proteins with peroxynitrite, which result in heterogeneous mixtures of nitrated protein due to lack of residue and site-specificity [12].

The need to generate homogenous and site-specific nitrated proteins has driven the development of improved systems for nitroTyr encoding [13,14]. For researchers new to GCE, selecting an appropriate system and set of expression conditions can be challenging. In this protocol, we focus on two broadly effective and well-validated systems derived from the *Methanocaldococcus jannaschii* tyrosyl pair (MjTyrRS/tRNA) [13] and the *Methanomethylophilus alvus* pyrrolysyl pair (MaPylRS/tRNA) [14], both of which have been engineered to support high-fidelity nitroTyr incorporation. While site-specific homogenous nitration is the goal, outcomes are often context-dependent and users may experience common GCE encoding issues observed under suboptimal GCE expression conditions, such as competition with release factor 1 (RF1) leading to truncated proteins [13], near-cognate suppression inserting natural amino acids at TAG sites [15], or chemical reduction of the encoded nitroTyr to 3-amino-tyrosine [16,17]. This protocol provides clear strategies for selecting appropriate cell lines, media formulations, and induction conditions that effectively mitigate these challenges.

Specifically, we present a detailed, step-by-step workflow for site-specific nitroTyr incorporation in *E. coli*, guiding users in the selection between release factor 1 (RF1)-containing [BL21(DE3)] and RF1-deficient [B95(DE3) Δ*A*Δ*fabR*] strains, media conditions that minimize misincorporation or chemical reduction, and appropriate use of either *Mj*- or *Ma*-derived GCE systems. While the protocol is demonstrated using superfolder GFP (sfGFP) as a control protein, it is broadly applicable to other recombinant proteins. With careful optimization based on the guidelines provided here, researchers can reliably express homogeneously nitrated proteins suitable for downstream functional, structural, or mechanistic studies. Importantly, all required expression strains and GCE machinery plasmids described here are available through the public repository Addgene.

## Materials and reagents

**Biological materials**

**Expression strains**

1. BL21(DE3) (ThermoFisher, catalog number: EC0114)

This *E. coli* strain is optimized for over-expression of target proteins driven by the T7 transcriptional promoter, which is commonly used in pET expression vectors. It carries a genomic copy of the T7 RNA polymerase gene under control of the *lacUV5* promoter; induction with IPTG triggers T7 polymerase expression, leading to efficient transcription of the target gene and high yields of recombinant protein. Because this strain retains release factor 1 (RF1, the protein that terminates translation at TAG stop codons), expression of proteins containing TAG-encoded noncanonical amino acids can result in both truncated and full-length proteins. To prevent co-purification of truncated protein with the desired full-length protein, the use of a C-terminal affinity tag is recommended.

2. B95(DE3) Δ*A*Δ*fabR* (Addgene, catalog number: 197934)

This strain is a fast-growing BL21(DE3) derivative that lacks RF1, the protein responsible for terminating translation at TAG codons, and carries a spontaneous mutation in the *fabR* gene [18]. To preserve cellular viability and reduce unintended readthrough in the absence of RF1, endogenous TAG stop codons in 95 genes were converted to TAA or TGA. Compared to standard BL21(DE3), this strain may be preferred for protein expression because RF1 deletion enhances TAG codon suppression by GCE machinery and minimizes production of truncated proteins caused by premature termination at TAG sites. However, this introduces another issue: near-cognate suppression. If the level of nitroTyr aminoacylated tRNA concentration is insufficient, native tRNAs in the cell (e.g., Gln-tRNA) can suppress TAG codons and result in the insertion of natural amino acids at the intended site of nitroTyr encoding. Thus, optimizing nitroTyr concentration, as well as choosing the right cell line and media combination, is crucial for faithful incorporation of nitroTyr in B95 cells. See below in “Choice of cell line, GCE machinery system and media” for more information.

**Plasmids**

1. pAJE-30-*Mj-*nitroTyr (Addgene, catalog number: 249488)

Machinery plasmid that enables encoding of nitroTyr at TAG codons. It expresses the *Methanocaldococcus jannaschii (Mj)-*TyrRS-nitroTyr “A7” [13] RS from a constitutive lpp promoter, as well as its cognate TAG codon-suppressing tRNA from a constitutive lpp promoter. This plasmid has spectinomycin resistance and contains a low-copy synthetic origin of replication [19,20] that is compatible with all standard origins, including ColE1/pBR322/pMB1, CDF, and p15A.

2. pAJE-30-*Ma-*nitroTyr (Addgene, catalog number: 249487)

Machinery plasmid that enables encoding of nitroTyr at TAG codons. It expresses the *Methanomethylophilus alvus (Ma)-*TyrRS-nitroTyr [14] RS from a constitutive GlnS promoter, as well as its cognate TAG codon-suppressing tRNA from a constitutive lpp promoter. This plasmid has spectinomycin resistance and contains a low-copy synthetic origin of replication [19,20] that is compatible with all standard origins, including ColE1/pBR322/pMB1, CDF, and p15A.

3. pET28-sfGFP WT (Addgene, catalog number: 85492)

Expresses wild-type sfGFP control protein with C-terminal His6 tag, under a T7 transcriptional promoter, kanamycin resistance, and pBR322 origin of replication. sfGFP is expressed by the addition of IPTG or lactose.

4. pET28-sfGFP TAG150 (Addgene, catalog number: 85493)

Same as above, except the *sfGFP* gene contains a TAG amber stop codon at site N150. The TAG codon is used to direct the translational encoding of nitroTyr.

5. pET28-(GOI) WT (you must clone)

Expresses your wild-type protein of interest (POI). You can clone the gene for your POI into the pET28 backbone by digesting with the restriction enzymes NcoI and XhoI, thus removing the gene for sfGFP. Your gene of interest (GOI) can then be put in using standard cloning techniques (ligation, Gibson Assembly, or SLiCE [21]).

6. pET28-(GOI) TAG (you must clone)

Expresses your POI with nitroTyr encoded at a TAG codon. You can clone the gene for your POI into the pET28 backbone by digesting with the restriction enzymes NcoI and XhoI, thus removing the gene for sfGFP. Your GOI can then be put in using standard cloning techniques (ligation, Gibson Assembly, or SLiCE [21]). The codon where you want to encode nitroTyr can be changed to a TAG codon using site-directed mutagenesis.

**Choice of cell line, GCE machinery system, and media**

Successful, efficient encoding and purification of nitroTyr into proteins is strongly dependent on three key experimental variables: cell line, growth media, and the choice of GCE machinery. Optimal expression and purification yield full-length nitrated protein, with >95% of protein molecules containing nitroTyr at the intended site. However, suboptimal conditions can lead to three common side products, as shown in Figure 1: (i) reduction of nitroTyr to 3-amino-tyrosine (aminoTyr) after successful incorporation, driven by the reducing environment of the cell; (ii) truncated protein, resulting from premature translation termination at TAG codons due to RF1; and (iii) misincorporation of glutamine via near-cognate suppression when the TAG codon is misread by endogenous tRNAs.

Each of these outcomes is highly context-dependent. The greatest variable is the reduction of nitroTyr to aminoTyr, since this is influenced by oxygen availability, media composition, the POI, and the site of encoding. AminoTyr cannot be encoded by the nitroTyr RS/tRNA pairs, but cellular reductases can convert some encoded nitroTyr protein sites to aminoTyr, provided encoding sites are accessible and expression conditions are limited in oxygen. To minimize reduction, cultures should be well-aerated using baffled flasks, antifoam agents, and high shaking speeds (250–300 rpm) during expression.

Truncation at TAG codons is inherent to RF1-positive strains such as BL21(DE3), but the effects of this issue can often be mitigated by using C-terminal purification tags, which allow for selective purification of full-length product. When C-terminal tagging is compatible with the POI, BL21(DE3) is a practical and effective option. By contrast, B95(DE3) Δ*A*Δ*fabR* is an RF1-deficient strain that enables truncation-free expression, supporting both N- and C-terminal tags without co-purification of prematurely truncated protein.

However, the B95(DE3) Δ*A*Δ*fabR* strain is more sensitive to expression inefficiencies: when intracellular nitroTyr is insufficient, near-cognate suppression may occur, most often resulting in glutamine incorporation at the intended nitroTyr site. In our experience, the likelihood of near-cognate suppression in B95(DE3) Δ*A*Δ*fabR* is strongly influenced by media composition because nitroTyr uptake is significantly more efficient in “defined” media compared to “complex” media (e.g., ZY) containing tryptone and yeast extract. Poor uptake of nitroTyr in “complex” media leads to low efficiency of nitroTyr tRNA aminoacylation, allowing endogenous tRNA suppression of the UAG codon to occur readily in B95(DE3) cells. In contrast, with BL21(DE3) cells, RF1 will dominate over near-cognate suppression, leading to translational termination instead of mis-encoding of canonical aminos at the UAG codon. With sfGFP proteins, we observe up to 40% misincorporation when nitroTyr-containing proteins are expressed in B95(DE3) Δ*A*Δ*fabR* cells using ZY “complex” media with either the *Mj* or *Ma* machinery systems. Switching to defined media effectively eliminates this issue and yields homogeneously nitrated, full-length protein in the B95(DE3) Δ*A*Δ*fabR* strain.

We have also observed that the *M. alvus* GCE system, while efficient, can be moderately toxic in B95 cells, likely due to background expression or metabolic burden. The *M. jannaschii* system is well tolerated in both BL21(DE3) and B95(DE3) Δ*A*Δ*fabR* strains. From a practical standpoint, ZY media is easy to prepare and suitable for routine use, whereas defined media requires more components and preparation time.

Given these considerations, we recommend the following strategies:

• When truncation is acceptable and C-terminal tags are compatible with your POI, we recommend the *M. alvus* GCE system in BL21(DE3) grown in ZY auto-induction media. This setup is robust, user-friendly, and produces high yields of homogeneously nitrated protein. The *Ma* nitroTyr GCE system can also be used to incorporate 3-chloroTyr, 3-bromoTyr, and 3-iodoTyr [13].

• When N-terminal purification tags are required, multi-site incorporation is desired, or truncation would compromise protein function, we recommend the *M. jannaschii* GCE system in B95(DE3) Δ*A*Δ*fabR*, grown in defined auto-induction media, to ensure full-length, site-specifically nitrated protein without misincorporation.

Table 1 summarizes the advantages and disadvantages of BL21(DE3) vs. B95(DE3) Δ*A*Δ*fabR* expression hosts and media combination to help users select the most appropriate strategy for their specific expression system.

**Table 1.** Summary of GCE system considerations when expressing proteins with nitroTyr

Feature/consideration	BL21(DE3)	B95(DE3) Δ*A*Δ*fabR*
RF1 status	RF1-positive → truncation possible	RF1-deficient → full-length expression only, no truncation
Recommended purification tag	C-terminal tag (to exclude truncated products)	N- or C-terminal tag
Recommended media	ZY auto-induction media	Defined auto-induction media
Misincorporation (i.e., near-cognate suppression)	Undetectable in all media	Undetectable in defined media; high in ZY media
Ease of media preparation	Easy (ZY-based media)	More labor-intensive (defined media)
GCE system recommendation	*M. alvus* (preferred), *M. jannaschii* (also compatible)	*M. jannaschii* (preferred due to lower toxicity)
Multi-site incorporation compatibility	Modest, 1–2 sites (due to RF1-mediated truncation)	Effective, up to 3 sites (no RF1 interference)

**Reagents**

**Essential reagents**

1. NaCl (e.g., VWR, catalog number: 97061-274)

2. α-D-glucose (e.g., VWR, catalog number: 97061-168)

3. α-lactose (e.g., RPI, catalog number: 26100-1000)

4. Glycerol (e.g., VWR, catalog number: BDH24388.320)

5. Na_2_HPO_4_ (e.g., VWR, catalog number: 97061-586)

6. KH_2_PO_4_ (e.g., VWR, catalog number: BDH9268)

7. NH_4_Cl (e.g., VWR, catalog number: 12125-02-9)

8. Na_2_SO_4_ (e.g., VWR, catalog number: 7757-82-6)

9. MgSO_4_ (e.g., VWR, catalog number: 7487-88-9)

10. CaCl_2_·2H_2_O (e.g., VWR, catalog number: 10035-04-8)

11. MnCl_2_·4H_2_O (e.g., VWR, catalog number: 13446-34-9)

12. ZnSO_4_·7H_2_O (e.g., VWR, catalog number: 7446-20-0)

13. CoCl_2_·6H_2_O (e.g., VWR, catalog number: 7791-13-1)

14. CuCl_2_ (e.g., VWR, catalog number: 10125-13-0)

15. NiCl_2_ (e.g., VWR, catalog number: 7791-20-0)

16. Na_2_MoO_4_·2H_2_O (e.g., VWR, catalog number: 10102-40-6)

17. Na_2_SeO_3_ (e.g., VWR, catalog number: 10102-18-8)

18. H_3_BO_3_ (e.g., VWR, catalog number: 10043-35-3)

19. FeCl_3_ (e.g., VWR, catalog number: 7705-08-0)

20. Kanamycin (e.g., VWR, catalog number: 75856-684)

21. Spectinomycin sulfate (e.g., VWR, catalog number: 89156-368)

22. Agar (e.g., VWR, catalog number: 97064-336)

23. Polypropylene glycol 2000 (PPG2000, antifoam) (e.g., Thermo Fisher Scientific, catalog number: AAL14699AP)

24. 3-Nitro-L-tyrosine (Thermo Fisher Scientific, catalog number: A11018)

**Reagents necessary only for ZY media**

25. Tryptone (e.g., VWR, catalog number: 97063-386)

26. Yeast extract (e.g., VWR, catalog number: 97064-368)

**Reagents necessary only for defined auto-induction media**

27. Glutamic acid, Na salt (e.g., VWR, catalog number: 56-86-0)

28. Aspartic acid (e.g., VWR, catalog number: 56-84-8)

29. Lysine-HCl (e.g., VWR, catalog number: 657-27-2)

30. Arginine-HCl (e.g., VWR, catalog number: 1119-34-2)

31. Histidine-HCl-H_2_O (e.g., VWR, catalog number: 5934-29-2)

32. Alanine (e.g., VWR, catalog number: 56-41-7)

33. Proline (e.g., VWR, catalog number: 147-85-3)

34. Glycine (e.g., VWR, catalog number: 56-40-6)

35. Threonine (e.g., VWR, catalog number: 72-19-5)

36. Serine (e.g., VWR, catalog number: 56-45-1)

37. Glutamine (e.g., VWR, catalog number: 56-85-9)

38. Asparagine-H_2_O (e.g., VWR, catalog number: 5794-13-8)

39. Valine (e.g., VWR, catalog number: 72-18-4)

40. Leucine (e.g., VWR, catalog number: 61-90-5)

41. Isoleucine (e.g., VWR, catalog number: 73-32-5)

42. Phenylalanine (e.g., VWR, catalog number: 63-91-2)

43. Tryptophan (e.g., VWR, catalog number: 73-22-3)

44. Methionine (e.g., VWR, catalog number: 63-68-3)

**Solutions**

**Essential solutions for all media**

1. LB/agar media (see Recipes)

2. 2× YT media (see Recipes)

3. SOC media (see Recipes)

4. Kanamycin stock (see Recipes)

5. Spectinomycin sulfate stock (see Recipes)

6. 25× M salts (see Recipes)

7. Trace metal stock solution (5,000×) (see Recipes)

8. 50× 5052 solution (see Recipes)

9. nitroTyr stock solution (see Recipes)

**Solutions necessary only for ZY media**

10. ZY media (see Recipes)

11. ZY non-inducing (ZY-NIM) and auto-inducing media (ZY-AIM) (see Recipes)

**Solutions necessary only for defined auto-induction media**

12. Aspartate [5% (w/v), pH 7.5] (see Recipes)

13. 18-amino-acid mix (25×) (see Recipes)

14. Defined NIM and AIM media (see Recipes)

**Recipes**

**1. LB/agar media**

**Table:** 

Reagent	Final concentration	Amount
Tryptone	1% (w/v)	5 g
Yeast extract	0.5% (w/v)	2.5 g
NaCl	1.0% (w/v)	5 g
Agar	1.5% (w/v)	7.5 g
H_2_O	n/a	To 500 mL
Total	n/a	500 mL

After mixing reagents thoroughly, autoclave on a standard liquid setting to sterilize. Note that the agar will not go into solution until autoclaved. After autoclaving, gently swirl the bottle to ensure molten agar is evenly mixed.

*Notes:*

*1. Store LB/agar bottle in a 65 °C oven and pour plates on an as-needed basis. LB/agar media can be stored in molten form for ~2 weeks, if sterility is maintained.*

*2. If an oven is not available, plates can be poured with antibiotics once LB/agar is sufficiently cooled to touch. Plates can be stored at 4 °C for up to a week.*

**2. 2× YT media**

**Table:** 

Reagent	Final concentration	Amount
Tryptone	1.6% (w/v)	16 g
Yeast extract	1.0% (w/v)	10 g
NaCl	0.5% (w/v)	5 g
H_2_O	n/a	To 1,000 mL
Total	n/a	1 L

After mixing the reagents thoroughly, autoclave on the standard liquid setting to sterilize. After autoclaving, allow it to cool to room temperature before use.

**3. SOC media**

**Table:** 

Reagent	Final concentration	Amount
2× YT media	n/a	49 mL
1 M MgSO_4_	10 mM	0.5 mL
40% (w/v) α-D-glucose	0.4% (w/v) or ~20 mM	0.5 mL
Total	n/a	50 mL

a. 1 M MgSO_4_ can be made by mixing 12.3 g of MgSO_4_·7H_2_O in water up to 50 mL total volume. Adjust the mass of MgSO_4_ accordingly if using a salt with a different hydration status.

b. 40% (w/v) α-D-glucose can be made by mixing 20 g of α-D-glucose with water up to 50 mL total volume. Mix thoroughly until glucose is dissolved. Gentle heating in a microwave may facilitate the dissolution of glucose.

c. Sterilize MgSO_4_, glucose, and 2× YT solutions individually by autoclaving. Allow each component to cool to room temperature and mix as indicated above. Maintain sterility while adding components together.

d. It is easy to contaminate SOC. We suggest breaking this into 5 × 10 mL aliquots before use or making smaller batches. If sterility is maintained, SOC can be stored at room temperature indefinitely. It can also be stored at -20 °C, but avoid repeated freeze/thaws.

**4. Kanamycin stock (10 mL)**

**Table:** 

Reagent	Final concentration	Amount
Kanamycin	50 mg/mL	0.5 g
H_2_O	n/a	To 10 mL
Total	n/a	10 mL

Sterilize by filtering with a 0.2 μm syringe-end filter. Store in 1 mL aliquots at -20 °C.

**5. Spectinomycin sulfate stock (10 mL)**

**Table:** 

Reagent	Final concentration	Amount
Spectinomycin sulfate	100 mg/mL	1 g
H_2_O	n/a	To 10 mL
Total	n/a	10 mL

Sterilize by filtering with a 0.2 μm syringe-end filter. Store in 1 mL aliquots at -20 °C.

*Note: Do not confuse spectinomycin with streptomycin. These antibiotics are not interchangeable.*

**6. 25× M salts**

**Table:** 

Reagent	25× concentration	Amount for 25×
Na_2_HPO_4_	0.625 M	88.7 g
KH_2_PO_4_	0.625 M	85.1 g
NH_4_Cl	1.25 M	66.9 g
Na_2_SO_4_	0.125 M	17.8 g
H_2_O	n/a	to 1 L
Total		1 L

Add the above components to a 2 L beaker containing a magnetic stir bar. Add water up to 900 mL and mix until all solutions have dissolved. Add the remaining volume of water to reach 1 L. Weights indicated are based on anhydrous salts. If using hydrated phosphate salts, adjust the weights accordingly to maintain the indicated molarities.

**7. Trace metal stock solution (5,000×)**

**Table:** 

Reagent	Concentration		Amount for individual 30 mL stocks
	**5,000×**	**1×**	
CaCl_2_·2H_2_O	20 mM	4 μM	8.82 g
MnCl_2_·4H_2_O	10 mM	2 μM	5.93 g
ZnSO_4_·7H_2_O	2 M	2 μM	8.62 g
CoCl_2_·6H_2_O	2 mM	0.4 μM	1.32 g
CuCl_2_	2 mM	0.4 μM	807 mg
NiCl_2_	2 mM	0.4 μM	777 mg
Na_2_SeO_3_	2 mM	0.4 μM	1.03 g
Na_2_MoO_4_·2H_2_O	2 mM	0.4 μM	1.45 g
H_3_BO_3_	2 mM	0.4 μM	371 mg
FeCl_3_	50 mM	10 μM	486 mg
H_2_O	n/a		To 30 mL

a. For each of the metals above (except FeCl_3_), make individual stock solutions using the indicated masses and dissolve in Milli-Q water up to 30 mL total volume. Autoclave each metal solution separately to sterilize. The FeCl_3 _must be dissolved in 0.1 M HCl up to 30 mL total volume and then filtered (through a 0.2 μm filter) to remove insoluble material and sterilize (do not autoclave).

b. Once all individual stock solutions are prepared, add 500 μL of each stock solution (except FeCl_3_) to 20.5 mL of sterile Milli-Q water. Then, add 25 mL of the FeCl_3_ solution. The total volume should be exactly 50 mL.

c. This stock solution might show minor precipitation over time, but it remains stable at 15–25 °C for years.

**8. 50× 5052 (500 mL)**

**Table:** 

Reagent	Final concentration	Amount
α-D-glucose	2.5% (w/v)	12.5 g
α-lactose	10% (w/v)	50 g
glycerol	25% (v/v)	125 mL
H_2_O	n/a	to 500 mL
Total	n/a	500 mL

Add the glucose, lactose, and glycerol components to roughly 300 mL of warm water in a 0.5 L beaker containing a magnetic stir bar. Mix until all solutions have dissolved. Additional heating may be required via microwave to encourage lactose dissolution. **Caution:** Remove the magnetic stir bar before microwaving. Once fully dissolved, add the remaining volume of water to reach 500 mL. Autoclave on liquid cycle to sterilize.

**9. nitroTyr stock solution**

**Table:** 

Reagent	Final concentration	Amount
nitroTyr	100 mM	226 mg
8 M NaOH	180 mM	225 μL
H_2_O	n/a	To 10 mL
Total	n/a	10 mL

Vortex the solution after combining to ensure all nitroTyr has dissolved. The solution can be split into 1 mL aliquots and stored at -20 °C for months. For optimal expressions, prepare the solution directly before use to avoid freeze/thaw cycles.

**10. ZY media**

**Table:** 

Reagent	Final concentration	Amount
Tryptone	1% (w/v)	10 g
Yeast extract	0.5% (w/v)	5 g
H_2_O	n/a	to 1 L
Total	n/a	1 L

Add the above components to a 1 L beaker containing a magnetic stir bar. Add water up to 900 mL and mix until all solutions have dissolved. Add the remaining volume of water to reach 1 L. Autoclave for sterilization.

**11. ZY-NIM and ZY-AIM**

**Table:** 

	ZY-NIM	ZY-AIM
**Reagent**	**Volume**	**Volume**
ZY media	47 mL	47 mL
MgSO_4_	0.1 mL	0.1 mL
25× M salts	2 mL	2 mL
50× 5052	–	1 mL
40% (w/v) α-D-glucose	0.625 mL	–
Trace metal (5,000×)	10 μL	10 μL
Total	50 mL	50 mL

a. When preparing media, dilute the concentrated components into ZY media. Do not mix concentrated stocks and then dilute with ZY media.

b. For BL21(DE3), final concentration for spectinomycin and kanamycin should be 100 and 50 μg/mL, respectively. For B95(DE3) expressions, the final concentration for spectinomycin and kanamycin should be 50 and 25 μg/mL, respectively.

c. Prepare immediately before use with sterile technique.

**12. Aspartate [5% (w/v), pH 7.5]**

**Table:** 

Reagent	Final concentration	Amount
Aspartate	5% (w/v)	50 g
H_2_O	n/a	To 1 L
Total	n/a	1 L

Mix by placing a suitable magnetic stir bar in a 2 L beaker and add 900 mL of water to the graduated cylinder. While stirring, add the appropriate amount of L-aspartic acid and adjust pH to 7.5 with 8 M NaOH. Add the remaining volume of H_2_O to bring the solution to a final volume of 1 L. Sterilize by autoclaving on a liquid setting.

**13. 18-amino-acid mix (25×) (1 L)**

**Table:** 

Reagent	Concentration		Amount for 25×
	**25×**	**1×**	
Glutamic acid, Na salt	200 μg/mL	8 μg/mL	5 g
Aspartic acid	200 μg/mL	8 μg/mL	5 g
Lysine-HCl	200 μg/mL	8 μg/mL	5 g
Arginine-HCl	200 μg/mL	8 μg/mL	5 g
Histidine-HCl-H_2_O	200 μg/mL	8 μg/mL	5 g
Alanine	200 μg/mL	8 μg/mL	5 g
Proline	200 μg/mL	8 μg/mL	5 g
Glycine	200 μg/mL	8 μg/mL	5 g
Threonine	200 μg/mL	8 μg/mL	5 g
Serine	200 μg/mL	8 μg/mL	5 g
Glutamine	200 μg/mL	8 μg/mL	5 g
Asparagine-H_2_O	200 μg/mL	8 μg/mL	5 g
Valine	200 μg/mL	8 μg/mL	5 g
Leucine	200 μg/mL	8 μg/mL	5 g
Isoleucine	200 μg/mL	8 μg/mL	5 g
Phenylalanine	200 μg/mL	8 μg/mL	5 g
Tryptophan	200 μg/mL	8 μg/mL	5 g
Methionine	200 μg/mL	8 μg/mL	5 g
H_2_O	n/a	n/a	To 1 L
Total	n/a	n/a	1 L

a. Add 800 mL of water to a 1 L beaker, then add 5 g of each amino acid while stirring with a magnetic stir bar. Since some amino acids have trouble dissolving in solution, warming the water prior to adding the amino acids can aid in the dissolution process. It may take several hours for each component to fully dissolve. Finally, bring the volume to 1 L with water.

b. Sterilize by filtration.

c. Aliquot 45 mL of 25× 18-amino acid mix into sterile 50 mL conical tubes.

d. Store aliquots at -20 °C. Thaw working aliquots as needed, which can be stored stably at 4 °C for several months, provided sterility is maintained.

**14. Defined NIM and AIM Media**

**Table:** 

	NIM	AIM
**Reagent**	**Volume**	**Volume**
Aspartate [5% (w/v) pH 7.5]	2.5 mL	2.5 mL
50× 5052	–	1 mL
18-amino-acid mix	2.0 mL	2.0 mL
25× M salts	2.0 mL	2.0 mL
MgSO_4_ (1 M)	100 μL	100 μL
Glucose [40% (w/v)]	6.25 mL	–
Trace metal solution (5,000×)	10 μL	10 μL
Sterile H_2_O	36.64 mL	41. 89 mL
Total	50 mL	50 mL

a. When preparing media, add the concentrated components to sterile H_2_O; do not mix concentrated stocks and then dilute with sterile H_2_O.

b. For BL21(DE3), final concentration for spectinomycin and kanamycin should be 100 and 50 μg/mL, respectively. For B95, the final concentration for spectinomycin and kanamycin should be 50 and 25 μg/mL, respectively.

c. Prepare immediately before use with sterile technique.

**Laboratory supplies**

1. 1.7 mL Eppendorf tubes (e.g., VWR, catalog number: 87003-294)

2. 100 mm plates (e.g., VWR, catalog number: 470210-568)

3. 500 mL graduated cylinder

4. 15 mL conical tubes (e.g., VWR, catalog number: 89126-798)

5. 50 mL conical tubes (e.g., VWR, catalog number: 89039-656)

6. 14 mL sterile culture tubes (e.g., VWR, catalog number: 60818-689)

7. 250 mL baffled flasks (e.g., VWR, catalog number: 89095-266)

8. Micro pipette tips 10 μL (e.g., VWR, catalog number: 76323-394)

9. Micro pipette tips 200 μL (e.g., VWR, catalog number: 76323-390)

10. Micro pipette tips 1,000 μL (e.g., VWR, catalog number: 76323-454)

11. Disposable PD-10 desalting column, with Sephadex G-25 resin, 1.0–2.5 mL samples (Cytiva, catalog number: 17085101)

12. TALON^®^ Superflow^TM^ (VWR, catalog number: CA71006-006)

13. Nalgene^®^ bottle-top sterile filter (Millipore Sigma, catalog number: Z358223-12EA)

## Equipment

1. Autoclave capable of sterilizing liquid media and culturing materials at 121 °C, with saturated steam pressure of 15 PSI

2. Expression equipment:

a. Static incubator for growing LB/agar plates (set to 37 °C) (e.g., VWR, catalog number: 97025-630)

b. Shaker incubator for growing liquid cultures (e.g., New Brunswick I26R, Eppendorf, catalog number: M1324-0004)

*Note: The shaker should be able to rotate at 250–300 rpm. Refrigeration is necessary for expressions below room temperature (<25 °C). Shaker deck should have clamps to hold 250 mL and 2.8 L Fernbach flasks.*

c. Optical density 600 nm spectrophotometer (e.g., Ultrospec 10, Biochrome, catalog number: 80-2116-30)

3. Fluorometer capable of reading sfGFP fluorescence (excitation 485 nm/emission 510 nm); handheld fluorometers work well for routine fluorescence reads (e.g., PicoFluor from Turner Biosystems)

4. Freezer (-20 °C) for storing plasmids and antibiotics (e.g., Fisher Scientific, catalog number: 10-549-264)

5. Ice machine (e.g., Fisher Scientific, catalog number: 09-540-003)

6. Water bath (42 °C)

## Procedure

This protocol was written for using chemically competent BL21(DE3) and B95(DE3) Δ*A*Δ*fabR* cells to transform plasmid DNA. Electrically competent cells can be used, but some considerations should be taken into account. Electrically competent cells are ~100× more competent than chemically competent cells, resulting in a lawn of colonies instead of individual colonies. Electrically competent cells must be diluted by the appropriate factor to achieve individual, same-sized colonies. We recommend following previously published protocols for preparing chemically [22] and electrically [23] competent cells.

Transformations must be performed fresh for every expression. Frozen glycerol stocks of BL21(DE3) and B95(DE3) Δ*A*Δ*fabR* containing expression plasmids should not be used to regrow cells for subsequent expression.

**A. (Day 1) Transformations**

1. Thaw aliquots of chemically competent BL21(DE3) or B95(DE3) Δ*A*Δ*fabR* on ice.

*Note: This protocol describes the expression of sfGFP WT and sfGFP TAG150. WT and TAG POIs can be expressed in a similar manner once cloned; however, we always recommend performing sfGFP in parallel with the POI expressions to verify media and expression conditions. Slight modifications may be necessary when adapting the protocol for your POI (see* ***Troubleshooting*** *section).*

2. Pre-chill two 1.7 mL Eppendorf tubes on ice for 5 min before adding any plasmids or cells. There will be one tube for each transformation (two for the sfGFP controls; pre-chill additional tubes for POI expressions).

3. Set a water bath to exactly 42 °C.

4. To each tube, add 1–2 μL of pAJE-30-nitroTyr-*Mj* or pAJE-30-nitroTyr-*Ma* and 1–2 μL of the desired target protein expression plasmid (sfGFP controls or the POI) into the pre-chilled 1.7 mL Eppendorf tube.

5. Add 50 μL of chemically competent cells to each tube. Mix DNA with cells by gentle pipetting or flicking the tube with your fingers.

6. Place the tubes on ice for 30 min.

7. Heat-shock the cells by placing the Eppendorf tube in the 42 °C water bath for 45 s. Make sure the lid is not submerged and does not come into contact with the water.

8. Immediately put the tubes back on ice for 2 min.

9. Add 500 μL of SOC media.

10. Let cells shake at 250 rpm for 2 h at 37 °C.

11. While cells are recovering, prepare the LB/agar plates, one for each of the sfGFP controls and one for each of the POI (WT and TAG).

a. Pour 50 mL of molten LB/agar into a sterile 50 mL conical tube. If using BL21(DE3) cells, add 50 μL of spectinomycin stock and 50 μL of kanamycin stock. Final concentrations will be 100 μg/mL spectinomycin and 50 μg/mL kanamycin. If using B95(DE3) Δ*A*Δ*fabR* cells, add 25 μL of spectinomycin and 25 μL of kanamycin. Final concentrations will be 50 μg/mL spectinomycin and 25 μg/mL kanamycin. Mix by gentle inversion, then pour 15–25 mL onto each plate.

b. Allow the agar to cool and fully solidify near a flame with the lid partially open. Agar should solidify within 20 min.

12. Plate the recovered cells onto the LB/agar plates.

a. To obtain a sufficient number of colonies, plate all cells. To do this, centrifuge the Eppendorf tubes at 3,000× *g* for 3 min, remove 400 μL of the supernatant by pipetting, resuspend the cell pellet in the remaining 100 μL by gentle pipetting, and then plate 100 μL of cells. Spread evenly over the agar plate with a sterile spreader or glass beads.

b. Let the plates dry with the lid partially open for approximately 20 min near a flame, then incubate the plates upside down overnight at 37 °C.

**B. (Day 2) Starter cultures**

**Figure 2.**
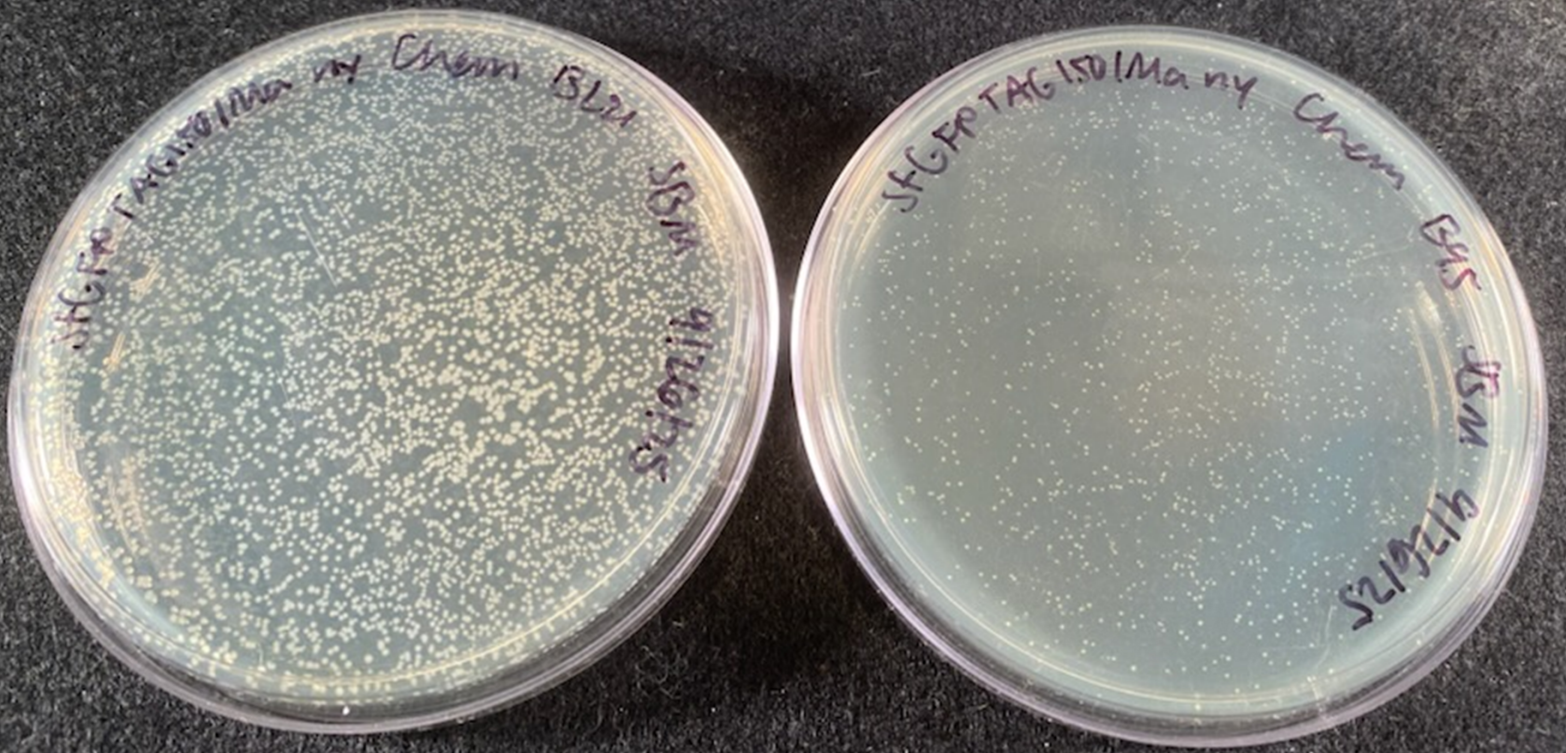
LB agar plates of transformed BL21(DE3) cells (left) and B95(DE3) Δ*A*Δ*fabR* cells (right) containing pET28-sfGFP TAG150 and pAJE-30-nitroTyr-*Ma*. The pET28-sfGFP TAG150/pAJE-30-nitroTyr-*Ma* transformation in B95(DE3) Δ*A*Δ*fabR* (right) tends to yield fewer and smaller colonies than the same transformation with BL21(DE3) cells (left). The slower growth of B95(DE3) Δ*A*Δ*fabR* cells with the *Ma* synthetase may be due to the GCE machinery in the recoded cells. The *Mj* synthetase does not appear to have this effect on cell growth in B95(DE3) Δ*A*Δ*fabR* cells. For more information on how to improve colony growth, see **Troubleshooting**.

1. Take the plates out of the 37 °C incubator and keep them at room temperature or in the refrigerator until the end of the day, when they will be used to inoculate overnight starter cultures. The difference in the number of colony-forming units between BL21(DE3) and B95(DE3) transformations can be seen in Figure 2; BL21(DE3) cells are generally more competent than B95(DE3) cells, and so more colonies are expected with BL21(DE3) transformations. Plates are stored for later use, since starter cultures should be inoculated at the end of the day, instead of at the start of the day, to avoid overgrowth.

2. Prepare 50 mL of non-inducing media (NIM) with the appropriate antibiotic concentrations for your cell line. See **Recipes** for the defined and ZY NIM recipes. Reminder: if you are expressing in BL21(DE3) cells, we recommend ZY NIM, and if you are expressing in B95(DE3) Δ*A*Δ*fabR* cells, we recommend defined NIM.

3. Label a 14 mL sterile culture tube with the correct plasmid combination. Add 5 mL of NIM to each culture tube.

4. To inoculate the starter cultures, scrape several dozen colonies from the LB/agar plate with a sterile pipette tip.

*Note: Expression levels can vary between different BL21(DE3)/B95(DE3) clones, which is why we recommend using several dozen colonies to inoculate starter cultures. This ensures that the amount of expressed protein is representative of the population average, making expressions considerably more reliable and consistent from one to the next. With the colony-loaded pipette tip in the sterile media, gently shake the cells off the pipette tip. Pipette any large clumps of cells up and down to disperse, if needed. Do not eject the tip into the media.*

5. Let cultures grow overnight (~12–18 h) at 37 °C while shaking at 250 rpm. Do not let starter cultures grow for more than 18 h.

**C. (Day 3) Expression**

1. Measure the OD_600_ of the starter cultures to ensure proper growth. For NIM, it should be between 1.5 and 4.

2. Prepare the nitroTyr stock solution as described in Recipes.

3. Prepare 50 mL of defined or ZY auto-induction media (AIM) as described in Recipes.

a. There will be three expression cultures for the sfGFP controls (sfGFP WT, sfGFP TAG150 with nitroTyr, sfGFP TAG150 without nitroTyr) and three for the POI (WT, TAG with nitroTyr, TAG without nitroTyr).

b. When doing multiple expressions, it is best to make a single batch of media, then aliquot or distribute 50 mL into each expression flask (for 50 mL expressions, use 250 mL baffled Erlenmeyer flasks). For example, if performing three 50 mL expressions, make 150 mL of media in one flask and then transfer 50 mL into each of the other two flasks. This ensures the media in all flasks have exactly the same components and concentrations, eliminating any variability in media preparation between cultures.

4. Add nitroTyr stock to the media of the “sfGFP TAG150 with nitroTyr” culture and the “POI TAG with nitroTyr” culture so that the final concentration in the expression is 0.5 mM.

5. Inoculate the expression cultures with a 1:100 dilution of the NIM starter cultures (e.g., if you are doing a 50 mL expression, inoculate with 0.5 mL of starter culture).

6. Add PPG2000 antifoam to allow proper aeration and prevent foaming. For a 50 mL expression, one small drop (approximately 10 μL) is sufficient.

7. Grow at 37 °C while shaking at 250 rpm for 24 h. If your POI requires a lower temperature, monitor the OD_600_ until it is 1.5, then lower it to the appropriate temperature. For BL21(DE3) cells, this should take 4–5 h. For B95(DE3) Δ*A*Δ*fabR* cells, this should take 5–6 h.

**D. (Day 4) Analysis of expression and cell harvesting**

1. After 24 h of expression, measure OD_600_ and fluorescence of sfGFP WT and sfGFP TAG150 (excitation/emission: 485/510 nm). Cells expressing sfGFP WT should be visibly green, regardless of cell line. If using BL21(DE3), sfGFP TAG150 without nitroTyr should not be yellow, while sfGFP TAG150 with nitroTyr should be yellow/green due to the incorporation of nitroTyr. If using B95(DE3) Δ*A*Δ*fabR*, sfGFP TAG150 without nitroTyr should be green due to near-cognate suppression, and full-length protein still being produced (without any nitroTyr encoded), while sfGFP TAG150 with nitroTyr should be yellow/green.

2. Harvest the cells by centrifuging at 5,000× *g* for 20 min at 4 °C. Pour off the supernatant and gently resuspend the pellet in an appropriate buffer that will be used for the lysis stage of the POI. The lysis buffer used will be dependent on the purification and downstream application of the protein. For His_6_-tagged, soluble (i.e., non-membrane-associated) proteins to be purified via TALON or Ni-NTA resin, a recommended “generic” resuspension/lysis/wash buffer is 50 mM Tris pH 7.5, 500 mM NaCl, 5 mM imidazole, and 10% (v/v) glycerol. Detergents and lysozyme are not required for cell lysis when using mechanical lysis methods such as sonication or microfluidization. The pH of the buffer can range from 7 to 8 for effective binding of the His_6_ tag to the metal affinity resin. We recommend NaCl concentrations ranging from 300 to 500 mM NaCl to minimize nonspecific ionic interactions between the POI and cellular proteins during purification. Glycerol is added to help stabilize proteins, while 5 mM imidazole is used to mitigate cellular proteins from binding nonspecifically to the IMAC resin. From here, the cell pellets may be frozen in liquid nitrogen and stored at -80 °C or directly purified. Typical yields from a 50 mL expression are approximately 10 mg for sfGFP WT and 1–5 mg for sfGFP TAG150.

## Validation of protocol

**Evaluation of nitroTyr encoding efficiency**

**Figure 3.**
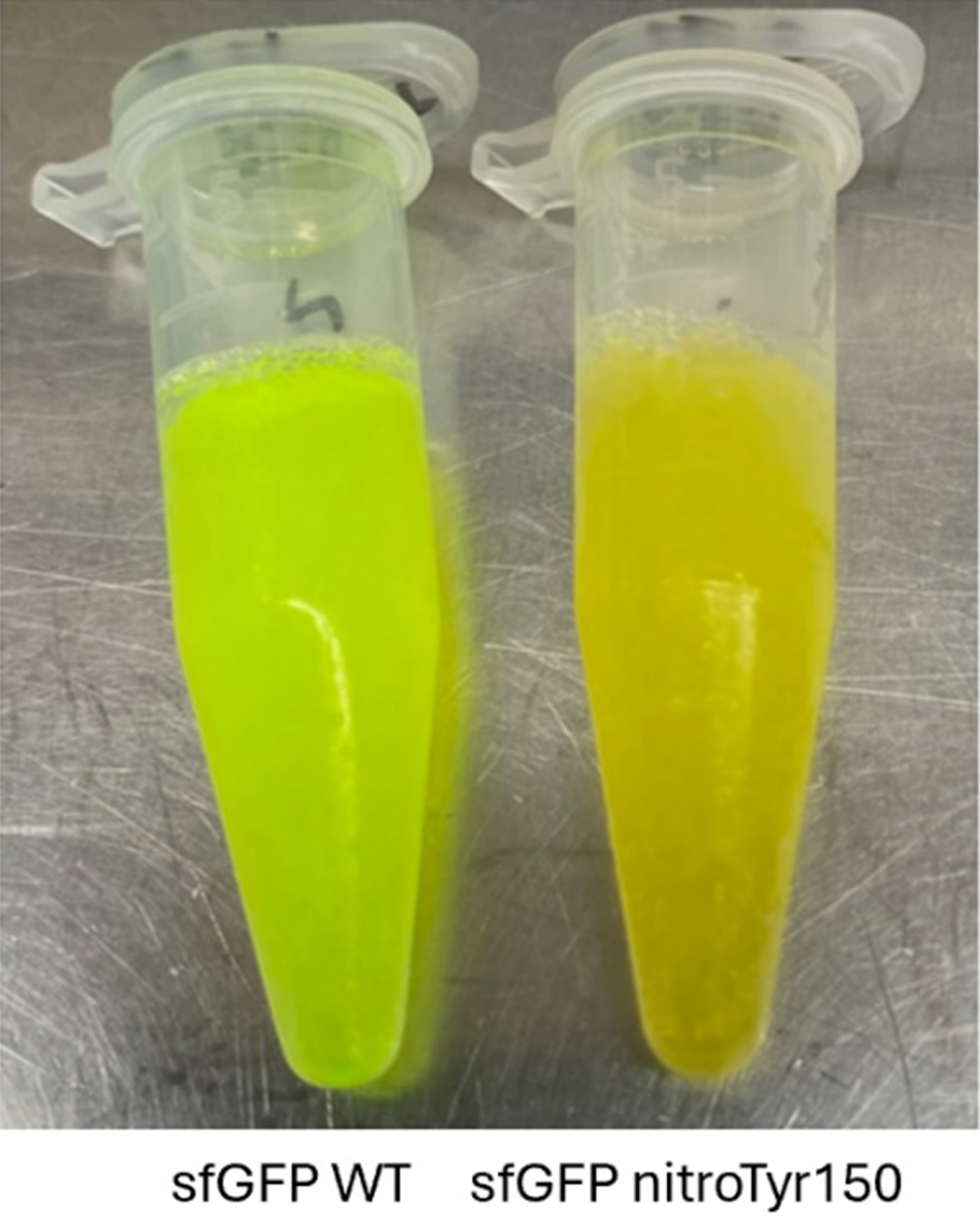
sfGFP WT and sfGFP nitroTyr150 after standard TALON purification

For this protocol, the sfGFP proteins were purified following standard (i.e., manufacturer recommendation) TALON resin protocols. Once purified, sfGFP WT should be green, and sfGFP nitroTyr150 should be yellow/green (Figure 3). A yellow color for your purified POI would indicate successful encoding of nitroTyr, but by itself does not confirm homogenous encoding. A lack of color in the purified POI with nitroTyr could indicate the nitroTyr on your protein was reduced to aminoTyr, near-cognate suppression occurred instead of nitroTyr (when using B95(DE3) cells), or the nitroTyr was encoded at a position where the phenolic oxygen is protonated, which quenches its color [24,25]. See **Troubleshooting** for more information.

Following protein purification, protein purity and encoding fidelity should be assessed. Protein purity is easily evaluated by SDS-PAGE gel analysis. The fidelity of nitroTyr encoding is evaluated by intact (i.e., whole-protein) mass spectrometry. In this protocol, intact mass spectrometry is used as the primary method because it enables assessment of the relative abundance of correctly encoded nitroTyr-containing protein compared with aminoTyr or mis-encoded species. For intact mass spectrometry, protein samples should be desalted into a mass spectrometry–compatible buffer to remove nonvolatile components. Here, sfGFP proteins were desalted into 50 mM ammonium bicarbonate using a PD-10 column according to the manufacturer’s protocols. Optimal sample preparation conditions may vary depending on instrumentation and should be determined in consultation with your mass spectrometry facility. Off-target incorporation at sites other than the intended TAG/UAG codon is highly unlikely using this amber suppression–based GCE approach, making the verification of site specificity less critical for routine sample analysis, but such MS/MS (i.e., tryptic digestion) confirmation of nitroTyr location may be required for publication purposes.

**Figure 4.**
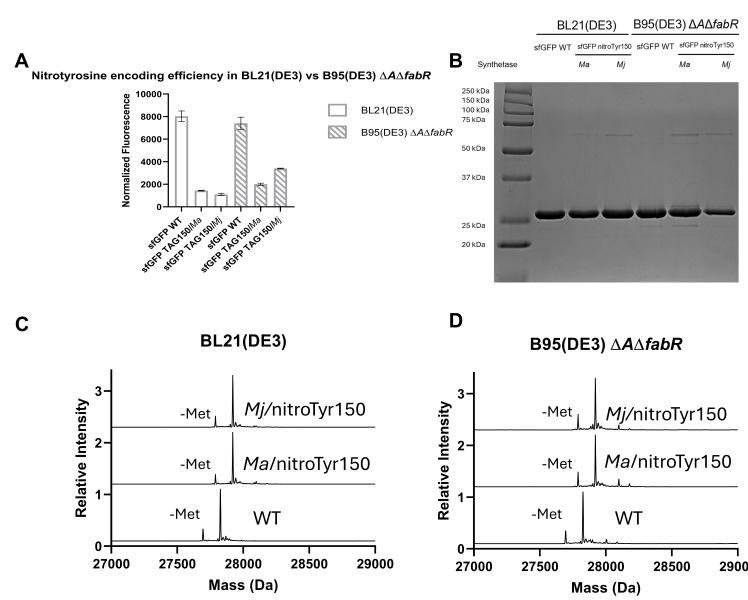
Evaluation of nitroTyr encoding efficiency and fidelity across different cell lines. (A) Normalized fluorescence for sfGFP WT and sfGFP TAG150 across the two cell lines with the different synthetases. BL21(DE3) cells were expressed in ZY media, and B95(DE3) Δ*A*Δ*fabR* cells were expressed in defined media. For GFP expressions, nitrated GFP will be approximately 25%–40% the fluorescence of WT GFP expressions. (B) SDS-PAGE gel analysis shows pure, full-length protein without any truncation products. (C) Mass spectrometry analysis of sfGFP WT and sfGFP TAG150 expressed in BL21(DE3) cells shows homogenous encoding, with both *Ma* and *Mj* GCE systems able to produce fully nitrated protein with no detected reduction or near-cognate suppression. The masses are listed in the following format: sfGFP measured (expected). sfGFP WT 27,827.3 Da (27,827.3 Da). sfGFP nitroTyr150/*Ma* 27,921.6 Da (27,921.4 Da). sfGFP nitroTyr150/*Mj* 27,921.3 Da (27,921.4 Da). (D) Mass spectrometry analysis of sfGFP WT and sfGFP TAG150 expressed in B95(DE3) Δ*A*Δ*fabR* cells shows homogenous encoding, with both *Ma* and *Mj* GCE systems able to produce fully nitrated protein with no detected reduction or near-cognate suppression. The masses are listed in the following format: sfGFP measured (expected). sfGFP WT 27,827.3 Da (27,827.3 Da). sfGFP nitroTyr150/*Ma* 27,921.3 Da (27,921.4 Da). sfGFP nitroTyr150/*Mj* 27,921.3 Da (27,921.4 Da).

The *Ma* and *Mj* synthetases both produce efficient, faithful encoding of nitroTyr across both cell lines, with 5–10 mg of sfGFP obtained from 50 mL expressions (Figure 4). If you observe a reduction in your POI, or if your cells have trouble growing, there are several things to consider, as discussed in the next section.

This protocol or parts of it has been used and validated in the following research article(s):

• Beyer et al.[13]. Overcoming Near-Cognate Suppression in a Release Factor 1-Deficient Host with an Improved Nitro-Tyrosine tRNA Synthetase. *J Mol Biol*. 432(16): 4690–4704. https://doi.org/10.1016/j.jmb.2020.06.014

• Avila-Crump et al. [14]. Generating Efficient Methanomethylophilus alvus Pyrrolysyl-tRNA Synthetases for Structurally Diverse Non-Canonical Amino Acids. *ACS Chem Biol.* 17(12):3458–3469. https://doi.org/10.1021/acschembio.2c00639

## General notes and troubleshooting

**General notes**

1. NitroTyr absorption properties: Proteins containing nitroTyr often appear yellow due to the characteristic absorbance of nitroTyr near 424 nm. Absorption at 424 nm occurs when the phenolic oxygen of nitroTyr is deprotonated; when protonated, the absorbance maximum shifts into the ultraviolet (~360 nm) [24,25]. The pKa of the phenolic oxygen of the free nitroTyr amino acid is ~7.1, so at physiologic pH and above, a significant portion of nitroTyr is deprotonated and yellow colored. At pH values well below 7, nitroTyr becomes protonated, giving a largely colorless appearance. When encoded into a protein, however, the local environment of nitroTyr can shift its pKa, such that it may become protonated (and therefore appear colorless) even in buffers above pH 7. For these reasons, color should be treated only as a preliminary qualitative indicator of encoding; accurate nitroTyr incorporation must be confirmed by mass spectrometry rather than visual inspection.

2. Alternate expression strategies: This protocol describes optimized conditions for the expression of sfGFP as a control protein. When expressing other, biologically relevant proteins, modifications to the expression conditions may be required to achieve efficient nitroTyr incorporation and acceptable protein yield. The following parameters should be considered:

a. Expression temperature. Some proteins do not express efficiently at 37 °C and may require lower induction temperatures. Optimal protein expression temperature is highly protein-dependent. A common effective strategy is to maintain cultures at 37 °C during the initial growth phase and reduce the temperature to the desired induction temperature once an OD_600_ of 1.0–1.5 is reached.

b. Duration of expression. The optimal length of expression may differ from the 24 h described in this protocol and is often linked to the chosen induction temperature. Expression time after temperature reduction is protein-dependent and should be empirically optimized, but in our experience, an expression period of at least 18 h following temperature reduction yields satisfactory results.

c. Solubilizing fusion tags. If the target protein exhibits low expression levels or poor solubility, fusion to an N-terminal solubilizing partner such as maltose-binding protein (MBP), glutathione S-transferase (GST), or small ubiquitin-like modifier (SUMO; bdSUMO) may improve folding and increase recoverable protein yield. To prevent interference with downstream applications, include a site-specific protease cleavage site (e.g., TEV or HRV 3C) between the fusion partner and the target protein to allow tag removal during purification. SUMO fusion proteins can be cleaved using their cognate SUMO proteases.

d. Manual induction. This protocol describes protein expression using auto-induction media. Manual induction can also be achieved by adding IPTG (final concentration in expression should be 0.5 mM) once an OD of 0.6–0.8 is reached. This may be preferred if the POI is unstable and requires a shorter expression time. In our experience, BL21(DE3) cells and 2× YT media work well for manual induction, but the overall protein yield is commonly lower with manual induction compared to the auto-induction methods described here.

**Troubleshooting**

**1. Reduction of nitroTyr to aminoTyr.** NitroTyr can be reduced to aminoTyr after it has been incorporated into a protein, due to the reducing environment of the cell [17,26]. Because aminoTyr does not absorb in the visible range, proteins that initially encoded nitroTyr but were reduced to aminoTyr during expression will lack the characteristic yellow color associated with nitroTyr-containing proteins. If the nitroTyr in your protein has been reduced to aminoTyr, indicated by a mass loss of 30 Da due to the loss of two oxygen atoms and gain of two hydrogen atoms, there are some strategies that may help mitigate this. In our experience, the amount of aeration during protein expression is the most important variable. To ensure maximal aeration, always use baffled culture flasks and shake at high rates. We recommend 250 rpm for most situations; however, increasing to 300 rpm or higher may help. However, a thick foam layer can form at high shaking speeds, reducing air access. Antifoam is essential to mitigate the foam layer and improve aeration. We have also observed that reduction can be dependent on the site of nitroTyr encoding. When possible, switching the location of the TAG site to another relevant site of nitration could make a notable difference in reduction.

**2. Improving cell growth.** You may experience slow-growing B95(DE3) Δ*A*Δ*fabR* cells when they are transformed with the *M. alvus* GCE platform. While *M. alvus*-derived synthetases are extremely useful for GCE due to their ability to suppress alternative stop codons and the fact that they are orthogonal to other commonly used GCE systems, they do have some drawbacks, the main one being their toxicity to B95(DE3) Δ*A*Δ*fabR* cells as seen by slower growth rates. As described under **Plasmids**, the pAJE origin of replication that is present on the machinery plasmids is a synthetic origin. There are two versions of the pAJE origin [19] that have been evaluated: one with a copy number of ~30 and one with ~90. To minimize toxicity, *M. alvus* synthetases with the low–copy number pAJE origin (30 copies) should be used. Users should ensure that the Addgene plasmid specified in this protocol is used, as *M. alvus* pAJE plasmids with higher copy-number origins (90 copies) are not compatible with B95(DE3) Δ*A*Δ*fabR* cells and can prevent cell growth. If there are no colonies/very few colonies present on the B95(DE3) Δ*A*Δ*fabR* plates 12–18 h after the transformations, the plates can be returned to the incubator for 6–8 h to continue growing until just prior to making the starter cultures. If the plates still have no colonies, perform fresh transformations with a lower antibiotic concentration. We recommend 25 μg/mL spectinomycin and 12.5 μg/mL kanamycin for the final concentration. If there are still no colonies, switch to the *M. jannaschii* synthetase.

## References

[1] Viappiani S. and Schulz R. (2006). Detection of specific nitrotyrosine-modified proteins as a marker of oxidative stress in cardiovascular disease. Am J Physiol Heart Circ Physiol. 290(6): H2167–H2168. 10.1152/ajpheart.00128 .2006 16489112

[2] Bruijn L. I., Beal M. F., Becher M. W., Schulz J. B., Wong P. C., Price D. L. and Cleveland D. W. (1997). Elevated free nitrotyrosine levels, but not protein-bound nitrotyrosine or hydroxyl radicals, throughout amyotrophic lateral sclerosis(ALS)-like disease implicate tyrosine nitration as an aberrant *in vivo* property of one familial ALS-linked superoxide dismutase 1 mutant. Proc Natl Acad Sci USA. 94(14): 7606 7611 7611. 10.1073/pnas.94 .14.7606 9207139 PMC23869

[3] Anjo S. I., He Z., Hussain Z., Farooq A., McIntyre A., Laughton C. A., Carvalho A. N. and Finelli M. J. (2024). Protein Oxidative Modifications in Neurodegenerative Diseases: From Advances in Detection and Modelling to Their Use as Disease Biomarkers. Antioxidants. 13(6): 681 10.3390/antiox13060681 38929122 PMC11200609

[4] Ferrer-Sueta G., Campolo N., Trujillo M., Bartesaghi S., Carballal S., Romero N., Alvarez B. and Radi R. (2018). Biochemistry of Peroxynitrite and Protein Tyrosine Nitration. Chem Rev. 118(3): 1338 1408 1408. 10.1021/acs.chemrev .7b00568 29400454

[5] Bandookwala M. and Sengupta P. (2020). 3-Nitrotyrosine: a versatile oxidative stress biomarker for major neurodegenerative diseases. Int J Neurosci. 130(10): 1047 1062 1062. 10.1080/00207454.2020 .1713776 31914343

[6] Radi R. (2004). Nitric oxide, oxidants, and protein tyrosine nitration. Proc Natl Acad Sci USA. 101(12): 4003 4008 4008. 10.1073/pnas.0307446101 15020765 PMC384685

[7] Radi R. (2013). Protein Tyrosine Nitration: Biochemical Mechanisms and Structural Basis of Functional Effects. Acc Chem Res. 46(2): 550 559 559. 10.1021/ar300234c 23157446 PMC3577981

[8] Cassina A. M., Hodara R., Souza J. M., Thomson L., Castro L., Ischiropoulos H., Freeman B. A. and Radi R. (2000). Cytochrome c Nitration by Peroxynitrite. J Biol Chem. 275(28): 21409 21415 21415. 10.1074/jbc.m909978199 10770952

[9] Balafanova Z., Bolli R., Zhang J., Zheng Y., Pass J. M., Bhatnagar A., Tang X. L., Wang O., Cardwell E., Ping P., .(2002). Nitric Oxide(NO) Induces Nitration of Protein Kinase Cε(PKCε), Facilitating PKCε Translocation via Enhanced PKCε-RACK2 Interactions. J Biol Chem. 277(17): 15021 15027 15027. 10.1074/jbc.m112451200 11839754

[10] Zhu P., Nguyen K. T., Estelle A. B., Sluchanko N. N., Mehl R. A. and Cooley R. B. (2023). Genetic encoding of 3‐nitro‐tyrosine reveals the impacts of 14‐3‐3 nitration on client binding and dephosphorylation. Protein Sci. 32(3): e4574. https://doi.org/10.1002/pro.4574 PMC9926477 36691781

[11] Franco M. C., Ye Y., Refakis C. A., Feldman J. L., Stokes A. L., Basso M., Melero Fernández de Mera R. M., Sparrow N. A., Calingasan N. Y., Kiaei M., .(2013). Nitration of Hsp90 induces cell death. Proc Natl Acad Sci USA. 110(12): e1215177110. https://doi.org/10.1073/pnas.1215177110 PMC3607042 23487751

[12] Reynolds M. R., Berry R. W. and Binder L. I. (2005). Site-Specific Nitration and Oxidative Dityrosine Bridging of the τ Protein by Peroxynitrite: Implications for Alzheimer's Disease. Biochemistry. 44(5): 1690 1700 1700. 10.1021/bi047982v 15683253

[13] Beyer J. N., Hosseinzadeh P., Gottfried-Lee I., Van Fossen E. M., Zhu P., Bednar R. M., Karplus P. A., Mehl R. A. and Cooley R. B. (2020). Overcoming Near-Cognate Suppression in a Release Factor 1-Deficient Host with an Improved Nitro-Tyrosine tRNA Synthetase. J Mol Biol. 432(16): 4690 4704 4704. 10.1016/j.jmb .2020.06.014 32569745 PMC7665880

[14] Avila-Crump S., Hemshorn M. L., Jones C. M., Mbengi L., Meyer K., Griffis J. A., Jana S., Petrina G. E., Pagar V. V., Karplus P. A., .(2022). Generating Efficient *Methanomethylophilus alvus* Pyrrolysyl-tRNA Synthetases for Structurally Diverse Non-Canonical Amino Acids. ACS Chem Biol. 17(12): 3458 3469 3469. 10.1021/acschembio.2c00639 36383641 PMC9833845

[15] O'Donoghue P., Prat L., Heinemann I. U., Ling J., Odoi K., Liu W. R. and Söll D. (2012). Near‐cognate suppression of amber, opal and quadruplet codons competes with aminoacyl‐tRNA^Pyl^ for genetic code expansion. FEBS Lett. 586(21): 3931 3937 3937. 10.1016/j.febslet .2012.09.033 23036644 PMC3488457

[16] Balabanli B., Kamisaki Y., Martin E. and Murad F. (1999). Requirements for heme and thiols for the nonenzymatic modification of nitrotyrosine. Proc Natl Acad Sci USA. 96(23): 13136 13141 13141. 10.1073/pnas.96 .23.13136 10557286 PMC23913

[17] Gerding H. R., Karreman C., Daiber A., Delp J., Hammler D., Mex M., Schildknecht S. and Leist M. (2019). Reductive modification of genetically encoded 3-nitrotyrosine sites in alpha synuclein expressed in E. coli. Redox Biol. 26: 101251 10.1016/j.redox .2019.101251 31226647 PMC6586993

[18] Mukai T., Hoshi H., Ohtake K., Takahashi M., Yamaguchi A., Hayashi A., Yokoyama S. and Sakamoto K. (2015). Highly reproductive Escherichia coli cells with no specific assignment to the UAG codon. Sci Rep. 5(1): e1038/srep09699. 10.1038/srep09699 PMC4434889 25982672

[19] Chaillou S., Stamou P. E., Torres L. L., Riesco A. B., Hazelton W. and Pinheiro V. B. (2022). Directed evolution of colE1 plasmid replication compatibility: a fast tractable tunable model for investigating biological orthogonality. Nucleic Acids Res. 50(16): 9568 9579 9579. 10.1093/nar/gkac682 36018798 PMC9458437

[20] Eddins A. J., Bednar R. M., Jana S., Pung A. H., Mbengi L., Meyer K., Perona J. J., Cooley R. B., Karplus P. A., Mehl R. A., .(2023). Truncation-Free Genetic Code Expansion with Tetrazine Amino Acids for Quantitative Protein Ligations. Bioconjugate Chem. 34(12): 2243 2254 2254. 10.1021/acs.bioconjchem .3c00380 PMC11641772 38047550

[21] Zhang Y., Werling U. and Edelmann W. (2012). SLiCE: a novel bacterial cell extract-based DNA cloning method. Nucleic Acids Res. 40(8): e55–e55. 10.1093/nar/gkr1288 PMC3333860 22241772

[22] Green M. R. and Sambrook J. (2020). The Inoue Method for Preparation and Transformation of Competent *Escherichia coli*:“Ultracompetent” Cells. Cold Spring Harb Protoc. 2020(6): pdb.prot101196. https://doi.org/10.1101/pdb.prot101196 32482900

[23] Zhu P., Mehl R. and Cooley R. (2023). Biosynthesis and Genetic Encoding of Non-hydrolyzable Phosphoserine into Recombinant Proteins in Escherichia coli. Bio Protoc. 13(21): e4861. 10.21769/bioprotoc.4861 PMC10632156 37969748

[24] De Filippis V., Frasson R. and Fontana A. (2006). 3‐Nitrotyrosine as a spectroscopic probe for investigating protein–protein interactions. Protein Sci. 15(5): 976 986 986. 10.1110/ps.051957006 16641485 PMC2242503

[25] Yokoyama K., Uhlin U. and Stubbe J. (2010). Site-Specific Incorporation of 3-Nitrotyrosine as a Probe of p*K* _a_ Perturbation of Redox-Active Tyrosines in Ribonucleotide Reductase. J Am Chem Soc. 132(24): 8385 8397 8397. 10.1021/ja101097p 20518462 PMC2905227

[26] Chen H. C., Chang C., Lin W., Cheng D. and Leong M. (2008). H_2_O_2_/Nitrite‐Induced Post‐translational Modifications of Human Hemoglobin Determined by Mass Spectrometry: Redox Regulation of Tyrosine Nitration and 3‐Nitrotyrosine Reduction by Antioxidants. ChemBioChem. 9(2): 312 323 323. 10.1002/cbic.200700541 18161731

